# Supplementary material for: CD32 captures committed haemogenic endothelial cells during human embryonic development
Source: Nat Cell Biol. 2024 Apr 9;26(5):719–30. doi: 10.1038/s41556-024-01403-0 (PMC11098737; doi:10.1038/s41556-024-01403-0)
Supplement: Supplementary file 1 — Reporting Summary [file 41556_2024_1403_MOESM1_ESM.pdf]

nature portfolio

Corresponding author(s): Ditadi Andrea

Last updated by author(s): Feb 20, 2024

Reporting Summary

Nature Portfolio wishes to improve the reproducibility of the work that we publish. This form provides structure for consistency and transparency in reporting. For further information on Nature Portfolio policies, see our Editorial Policies and the Editorial Policy Checklist.

Statistics

For all statistical analyses, confirm that the following items are present in the figure legend, table legend, main text, or Methods section.

n/a	Confirmed	
☐	☒	The exact sample size (*n*) for each experimental group/condition, given as a discrete number and unit of measurement
☐	☒	A statement on whether measurements were taken from distinct samples or whether the same sample was measured repeatedly
☐	☒	The statistical test(s) used AND whether they are one- or two-sided<br>*Only common tests should be described solely by name; describe more complex techniques in the Methods section.*
☐	☒	A description of all covariates tested
☐	☒	A description of any assumptions or corrections, such as tests of normality and adjustment for multiple comparisons
☐	☒	A full description of the statistical parameters including central tendency (e.g. means) or other basic estimates (e.g. regression coefficient) AND variation (e.g. standard deviation) or associated estimates of uncertainty (e.g. confidence intervals)
☐	☒	For null hypothesis testing, the test statistic (e.g. F, t, r) with confidence intervals, effect sizes, degrees of freedom and P value noted<br>*Give P values as exact values whenever suitable.*
☒	☐	For Bayesian analysis, information on the choice of priors and Markov chain Monte Carlo settings
☒	☐	For hierarchical and complex designs, identification of the appropriate level for tests and full reporting of outcomes
☒	☐	Estimates of effect sizes (e.g. Cohen's *d*, Pearson's *r*), indicating how they were calculated

Our web collection on statistics for biologists contains articles on many of the points above.

Software and code

Policy information about availability of computer code

Data collection	For RNA sequencing, total RNA from human embryonic samples was prepared using Clontech SMART-Seq v4 Ultra Low Input RNA kit and sequenced using Illumina HiSeq 4000 with 1x50 single reads. Total RNA from human pluripontet stem cell-derived cultures was prepared using ReliaPrep RNA Cell Miniprep System, RNA-Seq libraries were generated using the Smart-seq2 method and sequenced using Illumina 395 NovaSeq6000 1x100 single reads. Reads were aligned to GENCODE GRCh38 using STAR v2.7.6a with standard parameters.<br><br>For single cell RNA-sequencing, cells from day 8 differentiation culture condition were methanol-fixed as previously described (doi:10.1186/s12915-017-0383-5). Libraries were prepared following the manufacturer's instructions using the Chromium platform (10x Genomics, Pleasanton, CA) with the 3' gene expression (3' GEX) V3 kit, using an input of ~10,000 cells. Libraries were sequenced in paired end mode on a NovaSeq instrument (Illumina, San Diego, CA) targeting a depth of 50,000-100,000 reads per cell. Sequencing reads were processed and akigned to GRCh38 using the Cell Ranger softeware pipeline (v4.0.0)<br><br>Flow cytometric data was collected using BD FACSDiva or Beckman Coulter CytExpert.
Data analysis	For RNA sequencing datasets, raw reads quality control was accomplished using the FastQC tool (http://www.bioinformatics.babraham.ac.uk/projects/fastqc) and read trimming was performed using the Trim Galore software (https://doi.org/10.5281/zenodo.5127899) to remove residual adapters and low-quality sequences. Trimmed reads were aligned against the human reference genome (GRCh38) using STAR (Dobin et al., 2013; https://doi.org/10.1093/bioinformatics/bts635) with standard parameters. Uniquely mapped reads were then assigned to genes using the featureCounts tool from the Subread package (Liao et al., 2014; https://doi.org/10.1093/bioinformatics/btt656), considering the GENCODE primary assembly v.34 gene transfer file (GTF) as reference annotation for the genomic features. Gene count matrices were then processed by using the R/Bioconductor differential gene expression analysis package DESeq2 (Love et al., 2014; https://doi.org/10.1186/s13059-014-0550-8) applying the standard workflow. For the human embryos' dataset, a paired analysis was set up modeling gene counts

using the following design formula: ~donor + condition. Gene p-values were corrected for multiple testing using FDR. Genes with adjusted p-values < 0.05 were considered differentially expressed. For single cell RNA sequencing gene counts for each cell were quantified with the Cell Ranger 'count' command with default parameters. The human genome (GRCh38.p13) was used as the reference. The resultant gene expression matrix was imported into the R statistical environment (version 4.0.3) for further analyses. Cell filtering, data normalization, and clustering were carried out using the R package Seurat (Stuart et al., 2019; https://doi.org/10.1016/j.cell.2019.05.031) v3.2.2. For each cell, the percentage of mitochondrial genes, number of total genes expressed and cell cycle scores (S and G1 phase) were calculated. Cells with a ratio of mitochondrial vs. endogenous gene expression > 0.2 were excluded as putative dying cells. Cells expressing <200 or >6,000 total genes were also discarded as putative poorly informative cells and multiplets, respectively. Cell cycle scores were calculated using the 'CellCycleScoring' function that assigns to each cell a score based on the expression of the S and G2/M phase markers and stores the S and G2/M scores in the metadata along with the predicted classification of the cell cycle state of each cell. Counts were normalized using Seurat function 'NormalizeData' with default parameters. Expression data were than scaled using the 'ScaleData' function, regressing on the number of unique molecular identifier, the percentage of mitochondrial gene expression, and the difference between S and G2M scores. By using the most variable genes, dimensionality reduction was then performed with principal component analysis (PCA) by calculating 100 PCs and selecting the top 55 PCs. Uniform Manifold Approximation and Projection (UMAP) dimensionality reduction (McInnes et al., 2018) was performed on the calculated principal components to obtain a 2D representation for data visualization. Cell clusters were identified using the Louvain algorithm at resolution r = 0.6, implemented by the 'FindCluster' function of Seurat. To find the differentially expressed (marker) genes from each cluster, the 'FindAllMarkers' function (iteratively comparing one cluster against all the others) from the Seurat package was used with the following parameters: adjusted P values <0.05, average log FC >0.25, and percentage of cells with expression > 0.1. A comprehensive manual annotation of the cell types was performed using the previously obtained markers list. Differentially expressed genes between cells of cluster 11 against cells of clusters 0,1, and 2 and clusters 16 and 17 were determined by the 'FindMarkers' function using the following parameters adjusted P values <0.05, |average log FC| > 0, and percentage of cells with expression > 0. GSEA was then performed considering Gene Ontology (GO) Biological Process (BP) terms from the C5 collection of the Molecular Signatures Database (MSigDB version 7.2) using the R/Bioconductor package clusterProfiler51 (v 3.8.1, http://bioconductor.org/packages/release/bioc/html/clusterProfiler.html). ORA was computed on the significantly differentially expressed genes considering Gene Ontology (GO) Biological Process (BP) terms from the C5 collection of the Molecular Signatures Database (MSigDB version 7.2) and the Reactome Pathways Database using the R/Bioconductor package 51 (v 3.8.1). P-values were corrected for multiple testing using FDR and enriched terms with an adjusted p-value less than 0.05 were considered statistically significant. Barplot was constructed using the R package ggplot2 (https://ggplot2.tidyverse.org). Single-cell RNA-seq samples from the public dataset GSE162950 were retrieved and processed as described in Calvanese et al., 2022 (https://doi.org/10.1038/s41586-022-04571-x) . The 'DotPlot' function from the Seurat R package was used to construct a scorecard highlighting the expression pattern of selected cells with specific expression patterns. Pseudotime trajectory was constructed using Monocle3 (version 0.2.3) (https://cole-trapnell-lab.github.io/monocle3/). Expression and feature data were extracted from the Seurat object and a Monocle3 'cell_data_set' object was constructed. The processed data was normalized followed by Principal Component Analysis (PCA) analysis using the Monocle3 function 'preprocess_cds'. Dimensionality reduction was performed using the 'reduceDimension' function. Trajectory graph learning and pseudo-time measurement through reversed graph embedding were performed with 'learn_graph' function. Cells were ordered along the trajectory using the 'orderCells' method with default parameters. The 'plot_cells' function was used to generate the trajectory plots.
To corroborate the findings from Monocle3, a trajectory inference analysis was conducted using the dynverse workflow, a component of the R package dyno (version 0.1.2).The Dynbenchmark utility, which offers a comprehensive framework for selecting the most suitable trajectory inference method according to the available experimental data, was utilized via the 'guidelines_shiny()' function. Following these guidelines, the trajectory inference analysis was performed using the Partition-based graph abstraction (PAGA)-tree algorithm. Input data for dyno, including gene expression matrices, dimensionality reduction coordinates, clustering information, and cell metadata, were derived from Seurat output and processed using the 'wrap_expression()' function. The cell trajectory was subsequently calculated by dyno using the 'infer_trajectory()' function, employing the 'ti_paga_tree()' method. Trajectory paths and pseudotime values were visualized on UMAP coordinates through the 'plot_dimred()' function provided by dyno.
To investigate the role of the CD32 gene in HEC ontogeny, genetic knockouts were simulated using the CellOracle tool (version 0.12.0). CellOracle integrates a gene regulatory network (GRN) with pseudotime analysis to predict shifts in cellular identities resulting from gene perturbations. This tool simulates alterations in gene expression due to perturbations and compares these changes to the cell's developmental trajectory within the GRN. This comparison allows for the estimation of transition probabilities between different cell states along the pseudotime axis. Following this, CellOracle generates a transition trajectory graph, illustrating the potential shifts in cellular identities after perturbation. This analysis was performed in a Python (version 3.8) environment, using Jupyter notebooks. Single-cell RNA sequencing (scRNA-seq) data, initially processed with Seurat, were converted to AnnData format using the anndata2ri tool (https://github.com/theislab/anndata2ri), ensuring content preservation for subsequent analysis. The CellOracle object construction utilized this data. Highly variable genes, critical for downstream analysis, were identified using the scanpy.pp.filter_genes_dispersion() function from scanpy, specifying n_top_genes=3000. A preliminary gene regulatory network (GRN) was constructed using the oracle.get_links() function within the Oracle() class, based on ligand-receptor interactions from the CellTalkDB database. This base GRN was further refined by incorporating the CD32 gene and its interactors, as identified in the STRING database (https://string-db.org/). Pseudotime analysis was conducted using the Pseudotime_calculator() class, employing the PAGA method from scanpy and integrating it into the CellOracle framework. This analysis culminated in the creation of a pseudotime gradient vector field with the Gradient_calculator() class from CellOracle, depicting the normal developmental trajectory. Subsequently, in-silico perturbation of CD32 expression and simulation of resultant cell identity shifts were performed using the simulate_shift() and estimate_transition_prob() functions from the Oracle class. To compare the effects of CD32 perturbation with normal development, the Oracle_development_module() class was used to calculate Perturbation Scores (PS) by computing the inner product of the respective vector fields with the calculate_inner_product() function.

All flow cytometric data was analyzed using FlowJo (v10)

For manuscripts utilizing custom algorithms or software that are central to the research but not yet described in published literature, software must be made available to editors and reviewers. We strongly encourage code deposition in a community repository (e.g. GitHub). See the Nature Portfolio guidelines for submitting code & software for further information.

Data

Policy information about availability of data

All manuscripts must include a data availability statement. This statement should provide the following information, where applicable:

- Accession codes, unique identifiers, or web links for publicly available datasets
- A description of any restrictions on data availability
- For clinical datasets or third party data, please ensure that the statement adheres to our policy

All new gene expression analysis datasets are available in the Gene Expression Omnibus (GEO) under the accession number GSE199578 and GSE223223. Accession is currently private, protected by a password and is scheduled to be released upon publication.
GSE199578 is used in Fig 1b-e; Fig 2a; Ext. Data Fig. 1d-e; Ext. Data Fig. 2a.
The GSE223223 dataset contains:
Subseries GSE223221 (Ext. Data Fig. 4a)
Sample GSM6943617 (Fig 4a-e; Ext. Data Fig. 4b-g)
Single-cell RNA-seq samples from the public dataset GSE162950 were retrieved and processed as described in Calvanese et al., 2022 (https://doi.org/10.1038/s41586-022-04571-x).
Reads were aligned against the human reference genome (GRCh38) using STAR v2.7.6a with GENCODE GRCh38.p13 version 34
Scripts used for data analysis and for the generation of all the figures in the paper are available at this link http://www.bioinfotiget.it/gitlab/custom/scarfo_hec2023.

Human research participants

Policy information about studies involving human research participants and Sex and Gender in Research.

Reporting on sex and gender	The reference to human participants included in this study refers to the patients who donate aborted tissue after signing the consent form after voluntary interruption of pregnancy.<br>Sex of the fetuses was not determined nor collected. All information derived from human material is completely anonymous, so they cannot in any way generate sensitive personal data, which affect the patient's privacy.
Population characteristics	Human embryos are obtained immediately after voluntary terminations of pregnancy induced with the RU 486 antiprogestative compound. Embryos are collected in sterile cold physiological solution containing antibiotics. Embryonic age is estimated based on several anatomic criteria: number of somite pairs at Carnegie stages 9 to 11, limb bud shape and eye pigmentation at stages 12 to 15 and 16 to 17, respectively.<br>As most of the tissues were destined for cell culture and sorting, specimens were preferably harvested under pseudo-sterile conditions. Thus, infected samples or samples from parents at risk for infectious pathologies (HIV, CMV, Hep-B-C, etc.) were excluded. Abortions performed on minors will not be sampled.
Recruitment	The tissues were obtained from elective pregnancy terminations. The decision to terminate pregnancy had occurred prior to consent for tissue donation. The tissues were entrusted, respecting anonymity (without any identity), to the research team which assigned a simple registration code allowing neither identification nor return to the patient. The only data necessary for the researcher is the term of pregnancy (weeks of amenorrhea). All documents concerning the patients, including the signed information and consent form, are kept by the clinical department in the patient's clinical file. This is stated clearly in the consent form that the patient signs.No payment were made to donors and the donors knowingly and willingly consented to provide research materials without restrictions for research and for use without identifiers.
Ethics oversight	Human embryonic tissues employed for RNA sequencing, immunohistochemistry and immunofluorescence were obtained from voluntary abortions performed according to the guidelines and with the approval of the French National Ethics Committee. Written consent to the use of samples in research was obtained from patients. The study was approved by Ospedale San Raffaele Ethical Committee (TIGET-HPCT protocol) and by the Institutional Review Board of the French Institute of Medical Research and Health (Number 21-854). Human embryonic tissues employed for RNA sequencing, immunohistochemistry and immunofluorescence were obtained from voluntary abortions performed according to the guidelines and with the approval of the French National Ethics Committee. Written consent to the use of samples in research was obtained from patients. The study was approved by Ospedale San Raffaele Ethical Committee (TIGET-HPCT protocol) and by the Institutional Review Board of the French Institute of Medical Research and Health (Number 21-854).

Note that full information on the approval of the study protocol must also be provided in the manuscript.

Field-specific reporting

Please select the one below that is the best fit for your research. If you are not sure, read the appropriate sections before making your selection.

☒ Life sciences ☐ Behavioural & social sciences ☐ Ecological, evolutionary & environmental sciences

For a reference copy of the document with all sections, see nature.com/documents/nr-reporting-summary-flat.pdf

Life sciences study design

All studies must disclose on these points even when the disclosure is negative.

Sample size	Experiments on human embryonic samples were performed at minimum in triplicates when feasible, i.e. in all but one case: in fact, the isolation of CD32+ and CD32neg cells from human embryonic samples for functional analysis was performed in duplicate. Differentiation experiments were performed in triplicate and including further replicates (up to seven) when feasible. Bulk RNA sequencing was performed in triplicate, as prior experience instructs is sufficient for statistical power. No statistical method was used to pre-determine sample size but sample size and replication were determined by historical controls e.g. Ditadi et al, 2015 (https://doi.org/10.1038/ncb3161).Replication was consistent with our prior publications. Single cell RNA sequencing was performed in single replicate as is standard within the field.
Data exclusions	Differentiation resuls were excluded when internal control failed to produce hematopoietic progenitors.
Replication	Experiments on human embryonic samples were performed at minimum in biological triplicate as reported in all figure legends. The isolation of CD32+ and CD32neg cells from human embryonic samples for functional analysis was performed in biological duplicate. Differentiation experiments were performed at minimum in triplicate. The specific number of replicates are indicated in all figure legends. Two additional, separate hPSC lines were employed in Figure 3g.
Randomization	Experimental conditions were not randomized but covariates were controlled by equal distribution of sorted cells across controls and experimental conditions.
Blinding	Blinding of experimental conditions was not relevant as our studies do not require grading of the results.

Reporting for specific materials, systems and methods

We require information from authors about some types of materials, experimental systems and methods used in many studies. Here, indicate whether each material, system or method listed is relevant to your study. If you are not sure if a list item applies to your research, read the appropriate section before selecting a response.

Materials & experimental systems

n/a	Involved in the study	
☐	☒	Antibodies
☐	☒	Eukaryotic cell lines
☒	☐	Palaeontology and archaeology
☒	☐	Animals and other organisms
☒	☐	Clinical data
☒	☐	Dual use research of concern

Methods

n/a	Involved in the study	
☒	☐	ChIP-seq
☐	☒	Flow cytometry
☒	☐	MRI-based neuroimaging

Antibodies

Antibodies used	Anti-human uncoupled antibodies used for immunohistochemistry and immunofluoresce includes: anti-human CD34 (Beckman, QBEnd/10, 1:500), anti-human ACE (BB9, BD Biosciences, 557813, 1:50), anti-human CD32 (Biolegend, 303202, 1:750) and rabbit anti-human/mouse Runx1 (Abcam, ab92336, 1:100). Secondary biotinylated antibodies were: goat anti-mouse IgG (Jackson Immuno Research, 115-066-072, 1:1000) and goat anti-rabbit IgG antibody (Jackson Immuno Research, 111-066-144, 1:500). Double-immunofluorescence staining used the Dylight488 coupled streptavidin (Biolegend, 405218, 1:350) and the TSA Fluorescent Plus System. For hPSC and human embryonic sample FACS analysis antibodies include: CD184 BV421 (BD, 562448, 1:100), CD32 APC Cyanine 7 (BD, 303229), CD32 PE (BD; 303206), CD34 APC (Beckman Coulter, IM2472), CD34 PE Cyanine 7 (Biolegend, 343616, 1:400), CD34 PE Cyanine 7 (eBioscience, 25034942, 1:400), CD4 PE Cyanine 7 (BD, 560649), CD43 APC (BD, 560198), CD43 FITC (BD, 555475, 10:100), CD44 PE (Miltenyi, 130-113-342), CD45 APC Cyanine 7 (Biolegend, 368516, 1:100), CD45 FITC (Beckman Coulter, A07782), CD45 BV421 (Biolegend, 304032), CD5 PE (Biolegend, 300607, 5:100), CD56 BV421 (BD, 740076, 3:100), CD7 APC (BD, 561604), CD73 BV421 (BD, 562430, 3:100), CD8 PE (Biolegend, 31051), DLL4 APC (Biolegend, 346508, 1:100), DLL4 BV421 (BD, 744840, 1:100), anti-IgG1 PE (Southern Biotechnology, 1070-05), 7AAD (BD, 559925, 1:100), Fcr Blocking reagent human (Miltenyi, 130-059-901, 20:100), CD3 V550 (BD, 561416, 1:30), CD45RA APC Cyanine 7 (Biolegend, 304128, 1:100), CD27 PE Cyanine 7, (eBioscience, 25027942, 1:100), CD25 BV421 (BD, 582442, 1:50), TCRb (BD, 749196, 1:100), TCRgd (Beckman Coulter, B49176, 1:100), CD45 BUV395 (BD, 563792), CD8 PerCP-Cy5.5 (Biolegend, 344710, 1:100), CD4 BV605 (BD, 562658, 1:50), TCRVd1 (Beckman Coulter, B49309, 1:50), TCRVd2 (Beckman Coulter, IM1464, 1:50),<br>All the antibodies used for FACS analysis were used at 1:200 dilution unless differently specified.
Validation	All antibodies are commercially validated. Validation statements found on manifacturer's website indicate the following validations: ACE (human bone marrow); CD32, CD34, CD4, CD43, CD45, CD44, CD5, CD56, CD7, CD73, CD3, CD45RA, CD27, CD25, TCRab, TCRgd, CD8,CD4, TCRVd1, TCRVd2 (human peripheral blood), FcR Blocking reagent (THP-1 cell line), RUNX1 (Molt-4 cell line).<br>Each lot of an antibody is tested for conformance with charachteristics of a standard reagent and representative flow cytometric data is included in data sheets to demonstrate specificity and/or sensitivity to a relevant cell population.

Eukaryotic cell lines

Policy information about cell lines and Sex and Gender in Research

Cell line source(s)	WA01 (H1) and WA09 (H9) were obtained from WiCell Stemcell bank. OP9DLL4 and OP9DLL1 were generated as described in Schmitt et al 2002 (https://doi.org/10.1016/S1074-7613(02)00474-0), Mohtashami et al, 2010 (https://doi.org/10.4049/jimmunol.1000782). MS5-DLL4 were a kind gift from Dr. Tom Taghon who described their generation in Dolens et al, 2020 (https://doi.org/10.15252/embr.201949006)
Authentication	Cell lines were authenticated.
Mycoplasma contamination	H1, H9, OP9DDL4 and OP9DDL1 were tested negative for mycoplasma contamination.
Commonly misidentified lines (See ICLAC register)	No commonly misidentified lines in the ICLAC registry are used in this study.

Flow Cytometry

Plots

Confirm that:

☒ The axis labels state the marker and fluorochrome used (e.g. CD4-FITC).

☒ The axis scales are clearly visible. Include numbers along axes only for bottom left plot of group (a 'group' is an analysis of identical markers).

☒ All plots are contour plots with outliers or pseudocolor plots.

☒ A numerical value for number of cells or percentage (with statistics) is provided.

Methodology

Sample preparation	Day 8 cells were trypsinized for 8 minutes and washed in IMDM, 10% FBS, 10 ug/ml DNAse. They were further dissociated with Collagenase II for 30 minutes and washed in Stempro 34. Human embryonic tissues were dissociated for 30-60 minutes with Collagenase I or Collagenase/Dispase. All samples were stained in Stempro 34 medium.
Instrument	Cells were sorted with FACSAria II (BD). FACS-analysis were performed at FACS Canto (BD Biosciences) Cytoflex S or Cytoflex LX (both Beckman Coulter).
Software	BD FACS Diva and Coulter CytExpert were used for data acquisition and FLowJo was used for analysis.
Cell population abundance	Single cells or 100-30000 CD32+ or CD44+ cells were isolated for each WNTd experiment. 100-30000 CD32neg cells were isolated for each WNTd experiment.
Gating strategy	Viable cells were gated using FSC-A/SSC-A and doublets were removed using FSC-H/FSC-W ans SSC-H/SSC-W. Autoflorescent cells were removed using the PerCP channel. 7AAD or Maleimide stain was used on human embryonic sample to remove the dead cells. Representative flow plots and gating strategy demonstrated in Extended Data Figure 1c, 2c, 2d, 3c, 8a<br>Figure 2e is gated on CD34+CD43negCD45neg.<br>Figure 3a, 3f, 4f, 4g, Extended data 4d, Extended data 4e, Extended data 4f, Extended data 6n, Extended data 7d, Extended data 7g and Extended Data Figure 8b are gated on live cells.<br>Figure 3b, Extended Data Figure 3d and Extended data 7b are gated on CD34+CD43negCD184negCD73neg cells.<br>Extended Data Figure 3f on CD34+CD43neg cells.<br>Figure 3c, Extended Data Figure 4a and Extended Data Figure 4b are gated on CD34+CD43negCD184negCD73negDLL4neg cells.<br>Figure 3h is gated on CD45+CD56negCD7+CD5+ cells.<br>Extended Figure 3f is gated on CD45+GFPnegCD3+ cells<br>Extended Figure 3g is gated on CD45+CD56neg cells<br>Extended Figure 3h is gated on CD45+CD56negTCRgd+ cells

☒ Tick this box to confirm that a figure exemplifying the gating strategy is provided in the Supplementary Information.
